# How to Account for Asymmetries in Deliberative Dialogues: Comment on "Evaluating Public Participation in a Deliberative Dialogue: A Single Case Study"

**DOI:** 10.34172/ijhpm.2023.7701

**Published:** 2023-05-15

**Authors:** Jaime Jiménez-Pernett

**Affiliations:** ^1^International Health Department, Andalusian School of Public Health, Granada, Spain; ^2^Public Health Research Center of the University of Montreal (CReSP), University of Montreal, Montreal, QC, Canada; ^3^Biosanitary Research Institute of Granada (ibs.GRANADA), Granada, Spain

**Keywords:** Public Engagement, Deliberative Methods, Inequalities, Equity, Measurement, Power Imbalances

## Abstract

In health policy-making, various deliberative mechanisms can be used to engage the members of the public in exploring what might be a reasonable course of action. Scurr et al take power dynamics into consideration to analyse a deliberative dialogue involving stakeholders with diverse points of view. Given such asymmetries at play, the conclusions of deliberations could be biased. Scholars would benefit from guidance on designing and evaluating deliberative processes. This commentary aims to broadly reflect on the possible sources of power and information asymmetries in deliberative dialogues, and to bring the biographical resources approach to deal with such asymmetries.

 Scurr et al^1^ offer a valuable empirical contribution to public engagement research through the evaluation of a deliberative dialogue. It worth noting that the authors took into account the power dynamics, an understudied concern in the field. Therefore, research on deliberative democracy has influenced many stakeholder consultations by fostering settings in which citizens can exchange reasons with mutual respect and equality. In line with this, I draw on literature from that particular discipline for more ideas about how to characterise and deal with such power asymmetries.

## Revisiting the Underlying Concept of Deliberative Inequality

 First, I would highlight the value of making explicit the underlying conceptual assumptions guiding the constructs and measures used by scholars when assessing a deliberative dialogue. Scurr et al gave an in-depth account of the planning process and the development of the deliberative dialogue with an emphasis on the participation of professional stakeholders and members of the public or tenants. This distinction suggests power and communicative asymmetries among both groups of participants, bringing up the underlying concept of deliberative inequality. According to Bohman,^2^ inequalities within deliberative processes are generated in communicative interactions by social position, communicative, and political capacities of participants. Taking a closer look at such interactions for sharing and scrutinizing information is essential for reducing the influence of asymmetric power relations and dominant approaches in deliberative processes.

 When measuring inequalities in deliberative processes, a claim to equality is usually implicit, but enforcing a principle of fairness might be helpful in protecting the right of individuals to participate in decision-making processes. An equity approach calls for a ‘just distribution of power and resources in relation to social circumstances and recognizing systematic differences between members of different social groups.’^3^ Scurr et al paid attention to diversity of needs among participants and offered innovative arrangements to the ‘traditional deliberative dialogue process’ to enable inclusion of community tenants. These adjustments include an orientation meeting prior to deliberation or a relaxation room for those participants. These contributions are useful for those who wish to design inclusive public engagement.

 The concept of deliberative inequality is based on public engagement interactions while the equity approach relies on the researcher’s subjective judgment about whether gaps detected in deliberative processes or outcomes are unfair, making it possible to adjust deliberative processes to mitigate the exclusionary consequences of those gaps. In my opinion, it would be equally important to incorporate a variety of measures for specific inequalities (ie, age, skills, or social roles) and equity measures to rebalance process and outcomes.

## Deepening the Measurement of Deliberative Inequalities and Equity in the Process

 A second point is the focus on the exclusionary consequences of asymmetries in deliberation. In this regard, Young^4^ argues that people from ethnic minority groups, women and working class men would be disadvantaged in deliberative processes. Two forms of exclusion in the communicative processes between public members and institutional agents appears. On the one hand, external exclusion embraces ‘the many ways that individuals and groups that ought to be included are, purposely or inadvertently, left out of fora for discussion and decision-making.’

 I appreciated that Scurr et al made the participant recruitment criteria explicit, such as: ‘be familiar with the available resources, supports, and challenges faced,’ ‘have good communication skills,’ ‘represented diverse ages, genders, races, employment status, and disabilities.’^1^ However, the results are only comparing two stakeholder groups (professionals and tenants), without providing information on the other criteria for recruiting participants. However, the results are limited to comparing two groups of stakeholders (professional or tenant), while not drawing on the other criteria for recruitment. Some studies offer further measures that may be useful in taking into account asymmetries related to external exclusion. At first, assessing the risk of self-selection bias^5^ is important to encourage the inclusion of participants with more diverse degrees of involvement in the community, promoting a wider range of opinions. Similarly, a more detailed reporting of nonresponses during the recruitment process^6^ could better account for external exclusion in a deliberative process. This includes non-responses during: (*i*) sampling of potential participants, (*ii*) contacting them, and (*iii*) the responses from potential participants (declining the invitation, being unable to participate, or not attending the deliberative event).

 The second form of exclusion raised by Young^4^ is the internal exclusion, referring to ‘the ways that people lack effective opportunity to influence the thinking of others even when they have access to fora and procedures of decision-making.’ This form of exclusion concerns the ways in which people express their ideas, speak up and listen to the arguments of others in deliberation. It underlies the common assumption that equal opportunity to speak increases the likelihood that a diversity of perspectives will be heard.^7^ Scurr et al adopt a qualitative approach to assess how participants occupied the communication space. To illustrate, they report that ‘many participants [...] claimed to feel that tenants dominated small group discussions,’ but ‘they rarely expressed direct disagreement or introduced new ideas without framing of a [tenant] narrative.’ Here, numerical metrics would help to provide details on the flow of the discussions to make comparisons between the small groups and with the plenary session, as well as further analyses with other deliberative dialogues. A comprehensive review of previous work provides a synthesis of a wide range of measures for online deliberations.^8^ These range from the simple number of speaking turns or word counts^9,10^ to composite measures of the distribution of participation in deliberations.^11,12^ A challenge in using these measures to quantitatively address dominance in deliberations is to define thresholds of unacceptable inequality with respect to the contributions of an individual or group.^13^

## Moving Beyond the Comparison of Stakeholders’ Categories Through a Biographical Resources Approach

 The case study by Scurr et al highlights the ‘merits of including those with lived experiences in setting priorities and making decisions in their own community.’ However, the main analyses tend to focus on exchanges between tenants and professional stakeholders. This kind of dichotomous opposition is very common in the public engagement studies but can lead to reflect a limited diversity of views in dialogue, and at the extreme, may induce the understanding of positions in terms of groupthink and polarisation.^14^

 The use of the biographical resources framework^15^ can provide a path to overcome these analytical limitations through a more holistic approach. Rooted in the scholarship of social movements, this framework emphasizes the role of resource mobilization and the life course approach by four dimensions: (*a*) cognitive resources; (*b*) cultural resources; (*c*) relational resources; and (*d*) life experience resources. Beyond classical sociodemographic stratifying variables, such as gender, social class, or educational attainment, some scholars have considered the influence of cognitive resources on deliberation, arguing that ‘participants with a higher level of prior knowledge of the issue may have a broader argumentative repertoire, which may positively influence their deliberative behavior.’^9^ Similarly, relational resources and lived experiences could modulate attitudes towards the topic under discussion and influence how some participants attribute meaning to knowledge. This ‘argumentative influence of deliberative-skilled participants’^10^ could also be a source of asymmetry. Scurr et al address these biographical resources, both qualitatively and quantitatively, considering, for instance, the previous participatory research experience of tenants, the background of chronic illness or social isolation. These experiences during the lifetime of the tenant are relevant to understanding their engagement in deliberation. Other aspects that may influence knowledge or attitudes towards the issue of social environment in the housing complex have not been reported, such as formal membership in tenants’ associative movement or civic organizations or family composition, informal personal networks or household composition.

 Taking into account biographical resources of the participants offers promising conceptual and operational definitions and refines the way in which the multidimensional nature of participatory citizenship can be measured. To move forward, these issues must be integrated into the design and planning process of deliberative dialogues by adopting a diversity approach that balances the profiles of the recruited participants and the composition of the groups not only by structural social positions, but also by the biographical elements that shape interactions in deliberation.

## Balancing Power Dynamics Between Stakeholders Must Take Into Account Deliberative Asymmetries

 In view of the democratic public deliberation literature, accounting for asymmetries at the different stages of the deliberative processes appears to be a growing concern. As such, Scurr et al are to be praised for mobilising a number of constructs and unfolding measures for some asymmetries influencing deliberative process and outcomes. These contributions could support researchers and practitioners in improving the legitimacy and relevance of deliberative processes. This commentary provides some insight to further the discussion on methods to address inequalities in the evaluation of deliberative processes and to better reflect in design and research the capacities of participants to act on inequalities through biographical resources.

## Acknowledgements

 I would like to thank Pascale Lehoux, my Thesis Director, for her valuable input in shaping the ideas presented in this manuscript. I also extend my thanks to Clara Bermudez for her insightful comments on an draft version of this commentary.

## Ethical issues

 Not applicable.

## Competing interests

 Author declares that he has no competing interests.
